# Stretching the Facial Nerve for the Relief of Spasm of the Facial Muscles

**Published:** 1881-03

**Authors:** 


### II. STRETCHING THE FACIAL NERVE FOR THE RELIEF OF SPASM OF THE FACIAL MUSCLES.

Dr. Allen Sturge and Mr. Godlee presented a paper on this subject. The patient, a lady, aged 72, had been sent to Dr. Sturge by Mrs. Garrett-Anderson. She had enjoyed good health until the death of her husband, six years previously. After this, her nervous system suffered much; she had fits of depression and debility; and, before long, twitching began round the right eye, extending subsequently to all the muscles supplied by the right facial nerve. She had gone through various courses of treatment without result; and she finally consented to have the facial nerve stretched. The operation was performed by Mr. Godlee on July 20th, by means of an incision behind the ear, from the external meatus nearly to the angle of the jaw. The sterno-mastoid and the parotid gland were pulled in opposite directions, exposing the upper border of the digastric, close to which the nerve was found as it emerged from the stylo-mastoid foramen. The nerve was raised on a hook, and pulled with moderate force. After a few such pulls, the right side of the face was completely paralyzed. The wound was dressed antiseptically, and healed without the appearance of a drop of pus, or the slightest constitutional disturbance. The face remained paralyzed for two months; and for some days after the operation there was a good deal of pain on the right side, and also in different parts of the head, which returned at intervals during these two months. When seen on October 19th, three months after the operation, the face at rest was nearly symmetrical on the two sides; but there was still a good deal of deficiency of movement in the muscles on the right side. She was, however, rapidly improving, every week making a considerable difference. The operation had now been performed five times—three times in Germany by Baum, Schussler and Eulenberg; once in America by Dr. James J. Putnam; the present case being the first case of the kind in England. In all these cases there was temporary paralysis after the operation, varying from two weeks in Baum's case to five months in Eulenberg's. It was remarkable that in every case in which the facial nerve had been stretched for spasmodic tic, the operation had been successful; whilst in several cases of spasmodic affection of other parts, as of the arm, etc., the stretching of the nerves of the part had produced no good effect. In these latter cases, the spasm had usually been of an elaborate character, allied rather to chorea; whereas the former was a simple unco-ordinated spasm of all the muscles supplied by a single nerve. The latter character would indicate a lesion in the center from which the facial nerve took its immediate origin, *i. e.*, the medullary center; whereas the former would point rather to a lesion of the co-ordinating center higher up in the cerebro-spinal axis. The stretching of the nerve certainly produced an immediate effect upon the nerve trunk itself, and it probably also produced a remote effect upon the nerve centers, which effect would be greater upon the lower centers, such as that of for the facial nerve in the medullar, than in those further removed, which were supposed to have to do with co-ordination. In all cases were nerve-stretching was employed for spasms, it would be very important to note whether the spasm was of the simple or of the co-ordinated variety, with a view to ascertain its relative value in the two varieties. The operation was rendered difficult on the living subject by the depth of the nerve and the constant trickling of blood into the wound. The operator should, therefore, secure a good light and efficient assistance. The posterior or auricular vein might very likely be cut on the first incision, and the posterior auricular artery at any period during the dissection. No vessel of any consequence was, however, likely to be injured if the wound was not carried more deeply than the digrastric muscle; should this level be passed the surgeon would find himself in dangerous proximity to the internal jugular vein.—Dr. Buzzard inquired if the effects of pressure on different parts of the face had been tried. This treatment had been found to stop chronic spasm, as in a case under his own care some two years ago. In another case, an accumulation of cerumen in the external meatus set up, by irritation, neuralgic pains, which ceased on removal of the wax and frequent application of a constant current. Dr. Buzzard said he had, at the present time, a man in hospital who was still under treatment, and in whom the supraorbital nerve had been stretched for neuralgia.—Mr. Walsham said that early in the year he had stretched the infra-orbital nerve for severe neuralgia; there was no pain after the operation. He believed the nutrition of the nerve to be that chiefly affected in the operation; this would not influence the intracranial centers, and the separation of the supplying minute vessels from the trunk of the nerve, might sufficiently account for the effects produced. Dr. Sturge said pressure had been made on different parts of the face without effect, and the final operation was had recourse to when all other treatment failed.—Mr. Godlee observed that the stretching must affect both the sheath of the nerve and the vessels. He had another case in which the nerve was stretched the preceding Wednesday, and was succeeded by twitching on both sides of the face.—Mr. Croft related his experience of a case to which he was called by Leibrich in 1877, and in which he cut down to the infra-orbital nerve of a patient, aged 65, who had suffered from severe convulsive neuralgia, the pain radiating along the branches of the infra-orbital nerve. He removed about five-eighths of an inch of this, and stretched it well, until a sense of "giving" was felt in the canal. The operation was performed on Wednesday. On the following Monday the patient left for Adelaide, whence a letter was dispatched saying that, for a time, pain and muscular spasm persisted, and then subsided. Six months later, an attack of gout was followed by convulsive neuralgia, which also subsided. After another six months, however, the pain once more returned, and to it succeeded permanent relief. Mr. Croft felt at a loss to define the correct treatment for such cases, some recommending a sensory, and others a motor, nerve to be operated on. He did not believe the center of disease to be intracranial. He remembered a case where, following amputation below the knee, the exposed nerve-ends were irritated, and became bulbous. The nerve was cut down on and resected, the ends being well stretched until they "gave" above. The patient, who was a lunatic, and under constant observation in an asylum, had suffered no return of the pain.—*British Medical Journal.*

